# A juvenile mouse model of anti-N-methyl-D-aspartate receptor encephalitis by active immunization

**DOI:** 10.3389/fnmol.2023.1211119

**Published:** 2023-09-18

**Authors:** Shuyu He, Chongyang Sun, Qian Zhu, Lin Li, Jianyu Huang, Ge Wu, Yi Cao, Jianxiang Liao, Yi Lu, Qiru Su, Sufang Lin, Xiaopeng Ma, Cheng Zhong

**Affiliations:** ^1^Shenzhen Key Laboratory of Precision Diagnosis and Treatment of Depression, CAS Key Laboratory of Brain Connectome and Manipulation, The Brain Cognition and Brain Disease Institute, Shenzhen Institute of Advanced Technology, Chinese Academy of Sciences, Shenzhen-Hong Kong Institute of Brain Science-Shenzhen Fundamental Research Institution, Shenzhen, China; ^2^Department of Clinical Research, Department of Neurology, Surgery Division, Epilepsy Center, Shenzhen Children's Hospital, Shenzhen, China; ^3^Shenzhen Children's Hospital of China Medical University, Shenzhen, China; ^4^University of Chinese Academy of Sciences, Beijing, China

**Keywords:** juvenile mouse model, anti-NMDAR encephalitis, cognitive deficits, epilepsy susceptibility, active immunization

## Abstract

**Introduction:**

Anti-N-methyl-D-aspartate receptor (NMDAR) encephalitis is a common autoimmune encephalitis, and it is associated with psychosis, dyskinesia, and seizures. Anti-NMDAR encephalitis (NMDARE) in juveniles and adults presents different clinical charactreistics. However, the pathogenesis of juvenile anti-NMDAR encephalitis remains unclear, partly because of a lack of suitable animal models.

**Methods:**

We developed a model of juvenile anti-NMDAR encephalitis using active immunization with an amino terminal domain peptide from the GluN1 subunit (GluN1_356 − 385_) against NMDARs in 3-week-old female C57BL/6J mice.

**Results:**

Immunofluorescence staining suggested that autoantibody levels in the hippocampus increased, and HEK-293T cells staining identified the target of the autoantibodies as GluN1, suggesting that GluN1-specific immunoglobulin G was successfully induced. Behavior assessment showed that the mice suffered significant cognition impairment and sociability reduction, which is similar to what is observed in patients affected by anti-NMDAR encephalitis. The mice also exhibited impaired long-term potentiation in hippocampal CA1. Pilocarpine-induced epilepsy was more severe and had a longer duration, while no spontaneous seizures were observed.

**Conclusion:**

The juvenile mouse model for anti-NMDAR encephalitis is of great importance to investigate the pathological mechanism and therapeutic strategies for the disease, and could accelerate the study of autoimmune encephalitis.

## 1. Introduction

Antibody-mediated encephalitis is a severe inflammatory brain disorder whose pathogenesis and cure remains challenging (Dalmau and Graus, 2018). Anti-N-methyl-D-aspartate receptor encephalitis was first identified in 2007 by the discovery of antibodies against neuronal cell-surface NMDARs (Dalmau et al., 2007, 2008). To date, anti-NMDAR encephalitis is the most common autoimmune encephalitis (AE), accounting for about 80% of all confirmed cases (Guan et al., 2016; Xu et al., 2022). Other antibody-mediated AE are associated with antibodies against neuronal cell-surface proteins, ion channels, or other receptors such as the α-amino-3-hydroxy-5-methyl-4-isoxazolepropionic acid receptor (AMPAR) (Zhang et al., 2021; Seery et al., 2022). It is particularly concerning that anti-NMDAR encephalitis is increasingly being recognized in patients aged ≤ 18 years, accounting for nearly 65% of all cases (Gable et al., 2012). Adult and pediatric patients with NMDARE exhibit different manifestations (Florance et al., 2009). Pediatric patients show more neurological symptoms, including seizures, movement disorders, insomnia, irritability and confusion, whereas adults present more psychiatric symptoms (Matricardi et al., 2016; Gastaldi et al., 2018; Dalmau et al., 2019; Cellucci et al., 2020). In addition, according to retrospective studies and clinical cases reports, the disease has a strong female predominance, and the median age of the patients is 21 years (Titulaer et al., 2013). However, no juvenile animal models are available at present to further investigate the underlying mechanisms of AE.

Adult NMDARE animal models are classified into those derived from an active immunization strategy with a peptide from the amino-terminal domain of the GluN1 subunit (which is included in all NMDAR), and those derived from a passive immunization strategy with affected cerebrospinal fluid (CSF) (Wright et al., 2015; Bai et al., 2023). NMDARs, a type of ionotropic glutamate-gated receptors (iGluRs) mainly expressed in the hippocampus, cortex, and striatum; play important roles in synaptic development, synaptic plasticity, learning, memory and cognition (Paoletti et al., 2013; Karakas and Furukawa, 2014; Lee et al., 2014; Gibson et al., 2020). Anti-NMDAR encephalitis is characterized by the presence of immunoglobulin G (IgG) antibodies against the GluN1 subunit of the NMDARs in CSF (Gleichman et al., 2012; Hara et al., 2018; Guasp et al., 2020). Studies using the passive immunization mice model have revealed that NMDAR function is disrupted by the binding and cross-link of autoantibodies against the endogenous NMDARs (Rosch et al., 2018; Taraschenko et al., 2019; Mannara et al., 2020; Radosevic et al., 2022; Steinke et al., 2023). Although the investigations based on the passive immunization model provided valuable information on the pathogenicity of the antibodies, this model is unable to reproduce the pathological process of autoantibody production *in vivo* (Wright et al., 2015; Taraschenko et al., 2019; Steinke et al., 2023). Thus, active immunization in juvenile female mice could provide further insight into the inflammation process and pave the way for further studies that may lead to the development of better treatments.

Currently, there are three existing options for the development of active immunization models of NMDARE, which are herpes simplex virus (HSV) infection, holoprotein immunization, and synthetic peptide immunization. HSV infection can trigger antibodies against NMDARs, but it acts indirectly (Armangue et al., 2018; Linnoila et al., 2019). The holoprotein immunization model is technically difficult and lacks enough specificity, because it requires conformationally-intact and stabilized native-like GluN1-GluN2B heterotetramers in liposomes, whereas synthetic peptide immunization requires an amino terminal domain of GluN1 (Gleichman et al., 2012; Jones et al., 2019). The variability and diversity of antibodies induced by synthetic peptide are less pronounced, due to the small size of the peptide. In addition, the holoprotein immunization model is more dependent on T-cell involvement, whereas the synthetic peptide immunization model has been confirmed to involve B-cell-mediated pathology, which is consistent with clinical research (Liba et al., 2016; Scheibe et al., 2017; Wagnon et al., 2020; Jiang et al., 2022). Active immunization with the GluN1_356 − 385_ peptide that targeted the amino-terminal domain of the GluN1 subunit (GluN1_356 − 385_) has been reported in an adult model (Ding et al., 2021). Thus, we chose the synthetic peptide immunization strategy to establish a juvenile female mouse model for NMDARE.

In this study, we developed a juvenile NMDARE mouse model using 3-week-old female mice by administration of an amino terminal domain (ATD) peptide from the GluN1 subunit (GluN1_356 − 385_) against NMDARs. Then, we confirmed the induction of anti-NMDAR antibodies in the experimental animals by brain immunofluorence and HEK-293T staining. Moreover, we performed open field, elevated plus maze, novel object recognition, and three-chamber tests to evaluate the anxiety, locomotion, cognition and social behaviors displayed by the model animals. We also conducted patch clamp electrophysiological recording to measure synaptic transmission efficiency and synaptic plasticity of the CA1 hippocampal area. Finally, we used electrophysiological recording and behavioral tests to evaluate the epileptic susceptibility of the mice.

## 2. Methods

### 2.1. Animals

Juvenile female C57BL/6J mice (8–12 g; 3 weeks old) were purchased from the Guangdong Medical Laboratory Animal Center (Guangdong Province, China). All mice were bred at the Shenzhen Institute of Advanced Technology using specific criteria. Animals were housed under the following laboratory conditions: ambient temperature, 24 ± 1°C; humidity, 50–60%; 12-h light/dark cycle beginning at 8 a.m.; food and water *ad libitum*. All experiments were performed in accordance with protocols approved by the Ethics Committee for Animal Research from the Shenzhen Institute of Advanced Technology, Chinese Academy of Sciences (SIAT-IACUC-220217-NS-ZC-A2121).

### 2.2. Experimental group allocation

The female mice were randomly allocated to three experimental groups: naïve, sham, and NMDARE. The order of the mice in the different experimental groups was also determined randomly at the beginning of the protocol. Due to ethical considerations, each cage contained animals from the same group in order to avoid mixing potentially sick animals with animals with milder or absent disease and thus exposing them to direct competition with animals in a better health condition.

### 2.3. Peptide design

Previous reports have demonstrated that the N368/G369 amino acids on GluN1 are essential for the binding of antibodies to NMDARs and their subsequent effects. Active immunization with the GluN1_356 − 385_ peptide from the amino terminal domain (ATD) of GluN1 effectively induces pathogenic anti-GluN1 autoantibodies. In this study, we used the GluN1_356 − 385_ peptide (LQNRKLVQVGIYNGTHVIPNDRKIIWPGGE, Genscript) to conduct active immunization in the NMDARE group.

### 2.4. Active immunization

The naïve group was blank, without any administration. The NMDARE group were subcutaneously administered 200 μL of emulsion mixture (volume ratio 1:1) comprising peptide dissolved in ddH_2_O and complete Freund's adjuvant (CFA, Chondrex, #7001) containing 4 mg/mL of heat-inactivated Mycobacterium tuberculosis H37Ra. Each mouse was administered the peptide at a dosage of 200 μg/injection, with three injections in total. In the case of the sham group, the peptide solution was replaced with ddH_2_O. Emulsion administration was scheduled at Day 1 (D1), D4, and D7. The method of administration was to inject 50 μL of emulsion at four different sites on the back of each experimental animal close to both upper and lower limbs, with a total amount of 200 μL. To increase blood-brain barrier permeability and assist autoantibodies to attack the central nervous system, each animal was intraperitoneally injected with 200 μL of pertussis toxin (PTX, Sigma-Aldrich, P7208) diluted in ddH_2_O at the 0 and 48 h timepoint after each peptide injection, for a total of six injections.

### 2.5. Behavioral experiment paradigm

Mice were subjected to different behavioral tests in the following order: open field test (OFT), elevated plus maze (EPM), three chamber test (TC), and novel object recognition (NOR), starting on D14 after the first immunization. Cerebrospinal fluid and serum samples were collected from the mice on D18 after all behavioral tests had been completed, and brain perfusion was performed on the same day. All behavioral experiment results were analyzed using a video-imaging system (Noldus Information Technology, EthoVisionXT).

#### 2.5.1. Open field test

The experimental setup consisted of a 50 × 50 × 50 cm open-field behavior box and a digital camera. At the beginning of the experiment, the mice were placed in the center of the open field. The mice allowed to move freely in the behavior box for 10 min and a behavioral video was recorded for further analysis. Between each trial, the behavior chamber was cleaned with a 20% ethanol solution. The number of entries and time spent in the central area (25 × 25 × 25 cm), as well as the total distance of locomotion, were recorded and analyzed.

#### 2.5.2. Elevated plus maze

The experimental setup consisted of an elevated plus maze placed 50 cm above ground level, and a digital camera. The elevated plus maze was divided into three parts, two opposite open arms (25 × 5 cm), two opposite closed arms (25 × 5 × 20 cm), and the intersectional center area (5 × 5 cm). At the beginning of the test, the mice were placed in the center area and allowed to move freely for 5 min across the maze. Between each trial, the behavior chamber was cleaned with a 20% ethanol solution. The number of entries and time spent in each of the three parts (open arms, closed arms, and center area), as well as the total distance of locomotion, were recorded and analyzed.

#### 2.5.3. Novel object recognition

The experimental setup consisted of a 50 × 50 × 50 cm plastic behavior box, four objects with two different shapes, and a digital camera. Nozzle tipping within an area of 3 cm around the object was defined as active exploration of the object. Before the test, mice were allowed to move freely in the behavior box without any object for 10 min during three consecutive days. On the day of the test, two 10-min tests were performed. First, two identical objects were placed at opposite corners of the box (northwest and southeast), with the object placed more than 10 cm from the edge of the box. At the beginning of the test, the mice were placed in the center of the box and allowed to explore it freely for 10 min. After a 1-h interval, one of the two identical objects was replaced with a novel object of a different shape, and the mice were allowed to explore the box for additional 10 min. The frequency and time spent actively exploring each object, as well as the total locomotion distance, were recorded and analyzed (mice that did not explore both objects for a minimum of 20 s were excluded). The Discrimination Index (DI-NOR) was calculated according to the following formula:


$$\begin{array}{l} DI-NOR\\ =\frac{time\;exploring\;the\;novel\;object\;-\;time\;exploring\;the\;familiar\;object}{time\;exploring\;the\;novel\;object\;+\;time\;exploring\;the\;familiar\;object} \end{array}$$

#### 2.5.4. Three chamber test

The experimental setup consisted of a 60 × 40 × 30 cm divided in three-chambers, two cages, and a digital camera. The three chambers were connected by two doors that could be opened or closed. The day before the test, the mice were placed into the box and allowed to explore it freely for 30 min. On the day of the test, the test was divided into three stages. In the first stage, the doors connecting the three chambers of the box closed. The mice were placed in the middle chamber and allowed to explore it freely for 5 min. In the second stage, the doors connecting the chambers were open, and two cages were placed inside the box at opposite corners, with one cage containing an unfamiliar mouse. The mice were allowed to explore the entire apparatus for 10 min. In the third stage, a second unfamiliar mouse was introduced into the other cage, and the mice were allowed to explore the entire apparatus freely for 20 min. Both unfamiliar mice were of the same sex as the experimental subject, but they had had no previous contact. The stage period began when the mice left the cage area after the first contact with the unfamiliar mouse. The frequency and duration of the visits to each unfamiliar mouse, and the total distance in the third stage, were recorded and analyzed. The Discrimination Index (DI-TC) was calculated according to the following formula:


$$\begin{array}{l} DI-TC\;=\frac{time\;exploring\;unfamiliar\;mouse\;2\;-\;time\;exploring\;unfamiliar\;mouse\;1}{time\;exploring\;unfamiliar\;mouse\;1} \end{array}$$

### 2.6. CSF and serum collection

Anesthesia was induced using 1.2% isoflurane and maintained throughout the experiment using 0.8% isoflurane administered via a facial mask. After the head was fixed in a standard stereotaxic frame, the skin and the first layer of muscle were cut from ear to neck. Next, the second and third layers of muscle were gently pulled apart to expose the endocranium. A glass electrode was used to puncture the endocranium at an appropriate angle to avoid vessel puncture. The cerebrospinal fluid was automatically sucked into the glass electrode via siphonage. After 10–20 min, the glass electrode was pulled out. The CSF sample was transferred to an eppendorf tube and centrifuged at 600 rpm (Hengnuo, MiniStar7K) for 10 s. Each collection was performed with a new glass electrode. CSF samples were stored at −80°C for later use. Blood samples were collected directly into a 1.5 mL eppendorf tube and incubated at room temperature for ~20 min. The samples were then centrifuged at 3,000 rpm (Eppendorf, Centrifuge 5418) for 10 min, and the supernatant was collected and stored at −80°C for later use.

### 2.7. Histology

Human embryonic kidney 293 (GluN1-transfected HEK-293T cells) cells were transiently transfected with NMDAR subunit genes (NR1), as previously reported (Ding et al., 2021). After being cultured for 24 h, cells were fixed on coverslips with 4% Paraformaldehyde and preincubated with 10% normal goat serum in 0.3% phosphate buffered saline (PBS) with TWEEN-20 (PBST). Then they were incubated overnight at 4°C with serum collected either mice from the sham group or from the NMDARE group in 0.1% PBST. After washing with PBS, the cells were labeled with G@M 594 (Jackson ImmunoRearch, 115-585-003, 1:200 in PBS) and observed under a confocal laser scanning microscope (Zeiss, LSM880).

For immunocytochemistry, brain slices with thickness of 35 μm were preincubated with 10% normal goat serum in 0.3% PBST and placed on a shaker at room temperature for 1 h. After being washed with PBS three times, the slices were incubated with fluorescent secondary antibodies diluted in PBS (Jackson ImmunoRearch, 115-547-003, 1:200 in PBS) for 2 h to detect autoantibodies.

### 2.8. Enzyme-linked immunosorbent assay

The experimental procedure was carried out in strict accordance with the instructions from the ELISA detection kit (Jianglai Bio, JL20420). Concentrations were measured in the range of 0.25–8 ng/ml. Standards were prepared by diluting the sample 10-fold, and adding 50 μL of sample or standard to each well. Hundred miceroloter Streptavidin-horseradish peroxidase (HRP) working solution was added to each well, and the wells were incubated at 37°C for 60 min. Samples were washed with Washing Buffer five times, using 350 μL per well each time. Fifty microliter of Chromogen Solution A and 50 μL Chromogen Solution B were added to each well, and the wells were incubated at 37°C for 15 min in darkness. After the addition of 50 μL Stop Solution to each well, the plate was placed into a microplate reader (BioTek, Synergy H1) to measure absorbance at 450 nm.

### 2.9. Electrode fabrication and implantation

Microwire electrode arrays, each containing 10 stereotrodes (20 channels), were manufactured using formvar-coated nickel-chromium wires with a diameter of 17.78 μm (HFV insulation, California Fine Wire Company). Each stereotrode was threaded through a silica tube (Polymicro Technologies, TSP100170). Each stereotrode was wrapped around two adjacent pins of a standard electrode connector (Omnetics connector, A79026). Silver microwires (OD = 200 μm, 99.95% pure) were then soldered to four pins on the outer side of the connector as ground and reference, respectively. Acrylic resin was used for encapsulation. The electrode tips were plated with platinum to reduce impedance to 300–800 kΩ (at 1 kHz in PBS) before use. The electrode arrays were manufactured as previously described by Sun et al. (2022).

For electrode implantation, a neural electrode was inserted in the brain at the following stereotaxic coordinates after craniotomy: the tips of the stereotrodes at AP −2.00 mm, ML −1.80 mm, and DV −2.00 mm for dCA3 recording.

### 2.10. Hippocampal slice preparation

After completion of the behavioral testing, three groups of female C57BL/6J mice consisting of four naïve, six sham and four NMDARE mice were used for electrophysiological experiments. Two types of artificial cerebrospinal fluid (ACSF) were prepared before experiments. High glucose ACSF (206 mM Sucrose, 1.3 mM KCl, 1.25 mM NaH_2_PO_4_, 11 mM Glucose, 26 mM NaHCO_3_, 10 mM MgSO_4_, 1 mM CaCl_2_) was pre-cooled in ice 30 min prior to the experiment and continuously bubbled with 95% O_2_ and 5% CO_2_. Standard ACSF (119 mM NaCl, 2.5 mM KCl, 1.25 mM NaH_2_PO_4_, 11 mM Glucose, 25 mM NaHCO_3_, 15 mM HEPES, 1.5 mM MgSO_4_, 2.5 mM CaCl_2_) was incubated in a water bath at at 32°C, and oxygenated. The animals were decapitated, and the entire brain was rapidly removed from the skull and immersed in ice-cold high glucose ACSF for 1 min. Then the hippocampal tissue was cut into 350 mm thick slices and incubated in oxygenated standard ACSF continuously bubbled with 95% O_2_ and 5% CO_2_ at 32°C for 30 min. The slices were incubated at room temperature for at least 1 h before recording. Two different slices from a single animal were used in field excitatory post-synaptic potentials (fEPSPs) recording.

### 2.11. Electrophysiological recording

Brain slices were transferred to the recording chamber and continually perfused with oxygenated standard ACSF (~2.5 mL/min, 32–34°C). A concentric stimulating electrode was positioned in the stratum radiatum (SR) of area CA1 to stimulate the Schaffer collateral (SC) pathway, 200 μm laterally to the recording electrodes, and stimuli (current pulses ranging from 50 to 300 μA) were delivered. The fEPSPs were recorded using 2–3 MΩ glass microelectrodes (SUTTER, BF150-110-10) filled with standard ACSF positioned under visual control in the SR subfield of area CA1 of the hippocampal slices, parallel to thestratum pyramidale (SP). fEPSPs were evoked by delivering a 1 ms electrical pulse via an isolated stimulator box (ISO-Flex). Signals were amplified with an amplifier (Axon MultiClamp 700B) and digitized through an acquisition board (Axon Digidata 1550B1) at 10 kHz. This input intensity was utilized to stimulate the SC pathway every 10 s and six responses averaged each minute. The stimulus intensity was continually increased to create input-output (I-O) curves. Subsequently, pairs of stimuli were applied to the same pathway separated by pre-determined intervals (50 ms) in order to analyze paired-pulse facilitation (PPF). The threshold for inducing a long-term potential (LTP) was established prior to the recording experiments. The stimulus intensity that elicited 50% of the maximum fEPSP response was utilized as the baseline input stimulus. LTP induction was induced by a series of high-frequency stimuli (HFS). We adapted a theta-burst stimuli (TBS) comprising 100 pulses at 100 Hz, repeated four times (George et al., 2022).

Electrophysiological signals were recorded using a 64-channel neural acquisition processor (Plexon, Dallas, TX, United States). Neural electrophysiological data acquired in this study were sampled at 1 kHz. Synchronized mouse behavior was recorded using a digital video camera (Plexon, Dallas, TX, United States). The protocol for pharmacologically-induced epilepsy was as follows: At the beginning of the test, the mice were placed in the home cage, and local field potential (LFP) and video recording were started. Thirty minutes later, the mice were intraperitoneally injected with scopolamine (aladdin, S129958, 1 mg/kg) diluted in saline. After additional 30 min, the mice were intraperitoneally injected with pilocarpine (aladdin, P129614, 320 mg/kg) diluted in saline. After the pilocarpine injection, LFP and video recording were performed during three additional hours.

### 2.12. LFP data analysis

Data analyses were performed using Clampfit (Version 10.6), Offline Sorter (Offline sorter application version 4.6.0), NeuroExplorer (NeuroExplorer version 5.310), and a custom software written in MATLAB. All LFP signals were processed through a 1–80 Hz band-pass digital filter with a sampling frequency of 1 kHz. Recorded files were manually inspected and only noise-free signals were used for the analysis. A 50 Hz notch filter was applied to remove the power line noise. For data analysis, spectrogram was generated by MATLAB using a Hanning window cosine (window size = 1 s, overlaps = 0.5 s). Time segments in which the root mean square power was more than 2.5-fold higher than that at baseline in the control period were considered as putative seizure periods (window size = 1 s, bin = 20 min).

### 2.13. Statistics

Data were analyzed using two-tailed two-sample *t*-tests and two-way ANOVA with the GraphPad Prism 8 software. All data are presented as the mean ± SEM. Significance levels are indicated as follows: *p < 0.05, **p < 0.01, and ***p < 0.001. The statistical details are shown in the respective figure legends.

## 3. Results

### 3.1. Glun1_356–385_ active immunization induced anti-NMDAR autoantibodies in juvenile female mice

**Figure 1:**
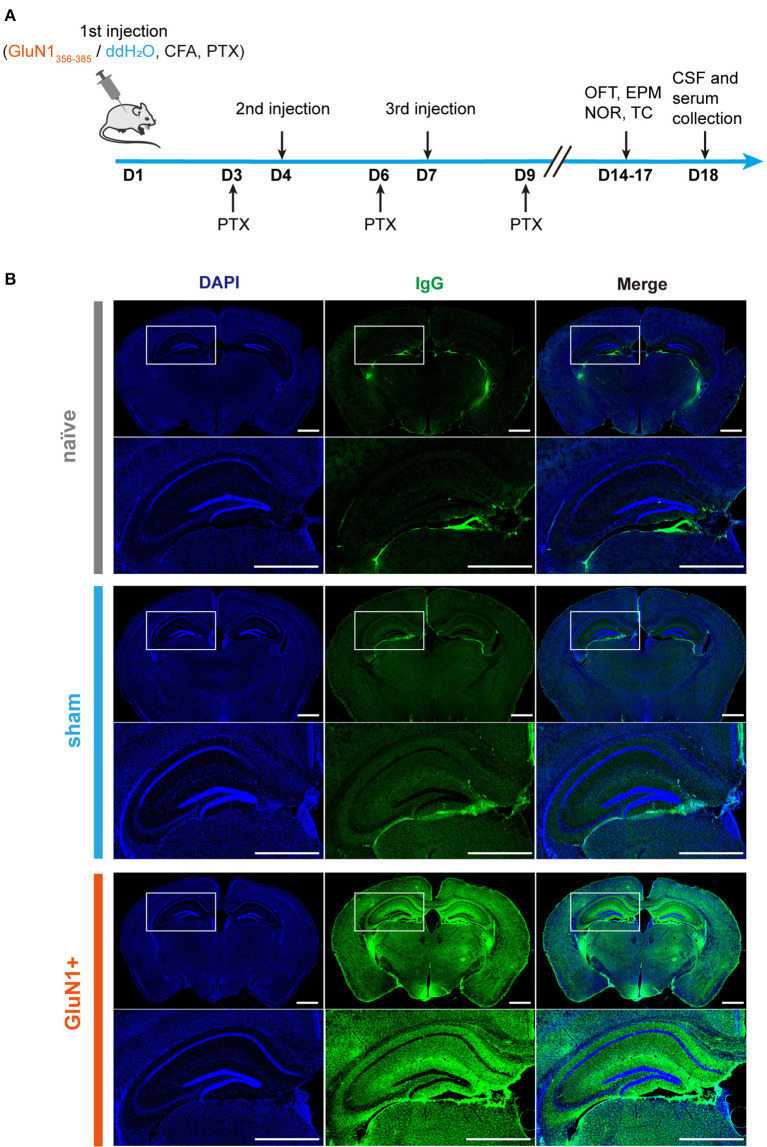
GluN1_356 − 385_ peptide induced IgG in the mouse brain. **(A)** The timeline of injections, behavioral tests, and sample collection. **(B)** Representative images of entire coronal brain slices and enlarged views of the dorsal hippocampal region showing the results of IgG immunofluorescence staining in the naïve, sham and GluN1+ groups (green: IgG, blue: DAPI). Scale bar, 1 mm.

To develop a mouse model for anti-NMDAR encephalitis using active immunization, female juvenile C57BL/6J mice were immunized with GluN1_356 − 385_ (Figure 1A). The administration of GluN1_356 − 385_ started on the third week after the birth, three injections were conducted with a 3-day interval. The experiments were conducted in NMDARE mice no more than 5 weeks old to ensure that the model was specific for juvenile NMDARE. In a previous study, the model mice were ~4 months old (Ding et al., 2021). Here, immunofluorescence staining for IgG showed strong autoantibody expression in the GluN1_356 − 385_-treated (GluN1+) group, located mainly in the hippocampus, cortex and hypothalamus (Figure 1B). These results show that autoantibodies were also produced partly in the sham group, maybe due to the blood brain barrier disruption derived from PTX treatment.

**Figure 2:**
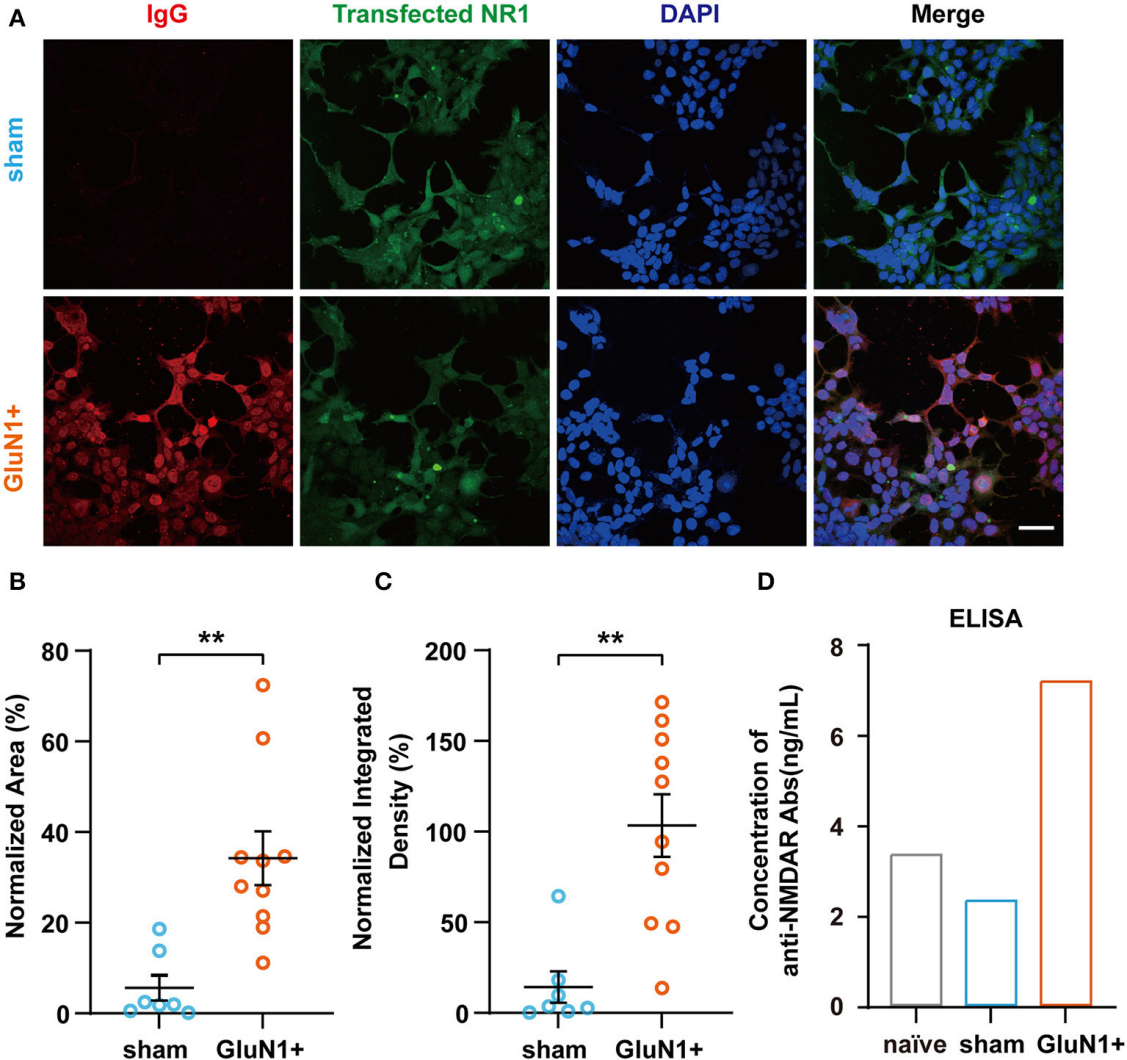
Confirmation of the presence of anti-NMDAR antibodies in GluN1+ mice. **(A)** Representative images of GluN1-transfected HEK-293T cells incubated with serum derived from animals from the sham or GluN1+ groups (red: IgG, green: NR1). Scale bar, 50 μm. **(B, C)** NR1 and anti-NMDAR antibody co-localization was quantified using the ratio of red to green fluorescence and the ratio of red to green integrated density (sham group, *n* = 7; GluN1+ group, *n* = 10). **p < 0.01, two-sample *t*-test. **(D)** Concentration of anti-NMDAR antibodies in each experimental group detected by ELISA. Samples were a mixture of serum from five animals in each group.

Since IgGs were also generated in the hippocampus of mice from the sham group, HEK-293T cells transfected with GluN1 subunits were used to determine whether the antibodies were GluN1-specific. Here, anti-NMDAR antibodies in the serum were detected. The result showed that GluN1 subunits on GluN1-transfected HEK-293T cells were co-localized with IgGs from the GluN1+ group, which suggests that the anti-NMDAR antibodies can be induced by GluN1_356 − 385_ (Figure 2A). The quantified data also suggested that the co-localization of generated the IgGs and NR1 was significantly increased in the GluN1+ group (Figures 2B, C). In addition, the results from the ELISA experiments confirmed that the levels of anti-NMDAR antibodies were higher in the in GluN1+ group compared to the sham group (Figure 2D). Taken these results together, it can be concluded that active immunization with GluN1_356 − 385_ is sufficient to induce anti-NMDAR antibodies in female juvenile C57BL/6J mice.

### 3.2. Locomotion and anxiety levels in juvenile female NMDARE mice

**Figure 3:**
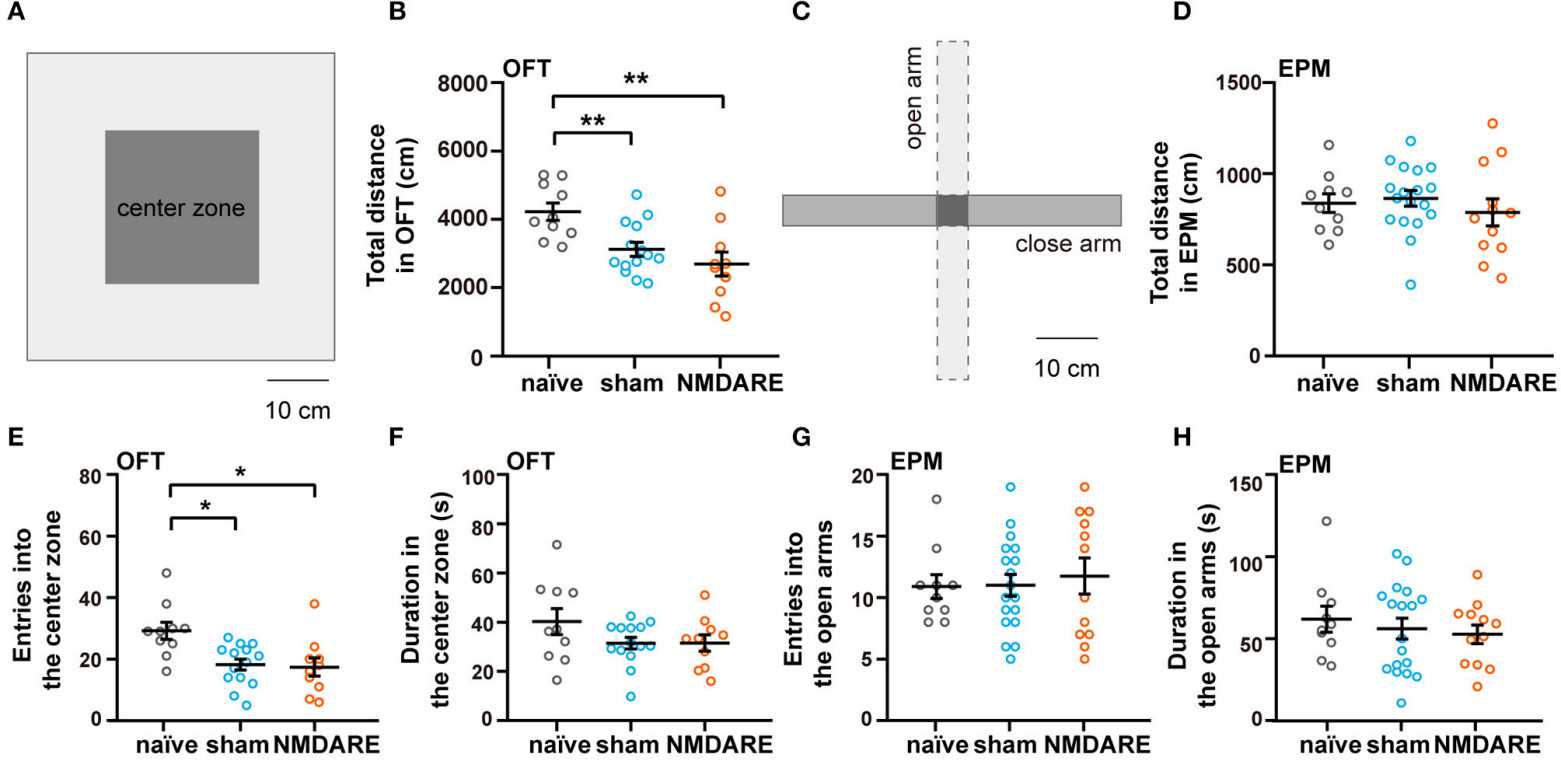
Assessment of anxiety level and locomotion activity of NMDARE mice via the open field and elevated plus maze tests. **(A)** Diagram of experimental setup for the OFT. Scale bar, 10 cm. **(B)** Total travel distance recorded during the OFT. **(C)** Diagram of experimental setup for the EPM test. Scale bar, 10 cm. **(D)** Total travel distance recorded during the EPM test. **(E)** Number of entries into the central zone during OFT. **(F)** Total time spent in the central zone during the OFT. **(G)** Number of entries into the open arms during the EPM test. **(H)** Total time spent in the open arms during the EPM test. OFT: naïve group, *n* = 10 mice; sham group, *n* = 14 mice; NMDARE group, *n* = 10 mice. EPM: naïve group, *n* = 10 mice; sham group, *n* = 18 mice; NMDARE group, *n* = 12 mice. *p < 0.05, **p < 0.01, two-sample *t*-test.

To investigate the effects of anti-NMDAR antibodies on behavioral phenotypes in NMDARE mice, EPM and OFT were performed to assess anxiety levels and locomotion activity. In the OFT, movement was significantly decreased in both the sham group and the NMDARE group, whereas it did not change in the EPM (Figures 3A–D). Mice from the sham group and NMDARE group showed decreased entries into the central zone during the OFT (Figure 3E). However, neither group exhibited significant changes in the time spent in the central zone (Figure 3F). Moreover, the number of entries and the time spent exploring the open arms in the EPM showed no significant changes in the sham group on in the NMDARE group (Figures 3G, H). Notably, there were no significant differences between the NMDARE and the sham groups for any of those behavioral parameters. In summary, this result demonstrated that the active immunization induced juvenile female NMDARE mice showed locomotion deficiency compared with naïve group, while they did not change anxiety levels and locomotion compared with sham group.

### 3.3. Cognition impairment and sociability reduction in juvenile female NMDARE mice

**Figure 4:**
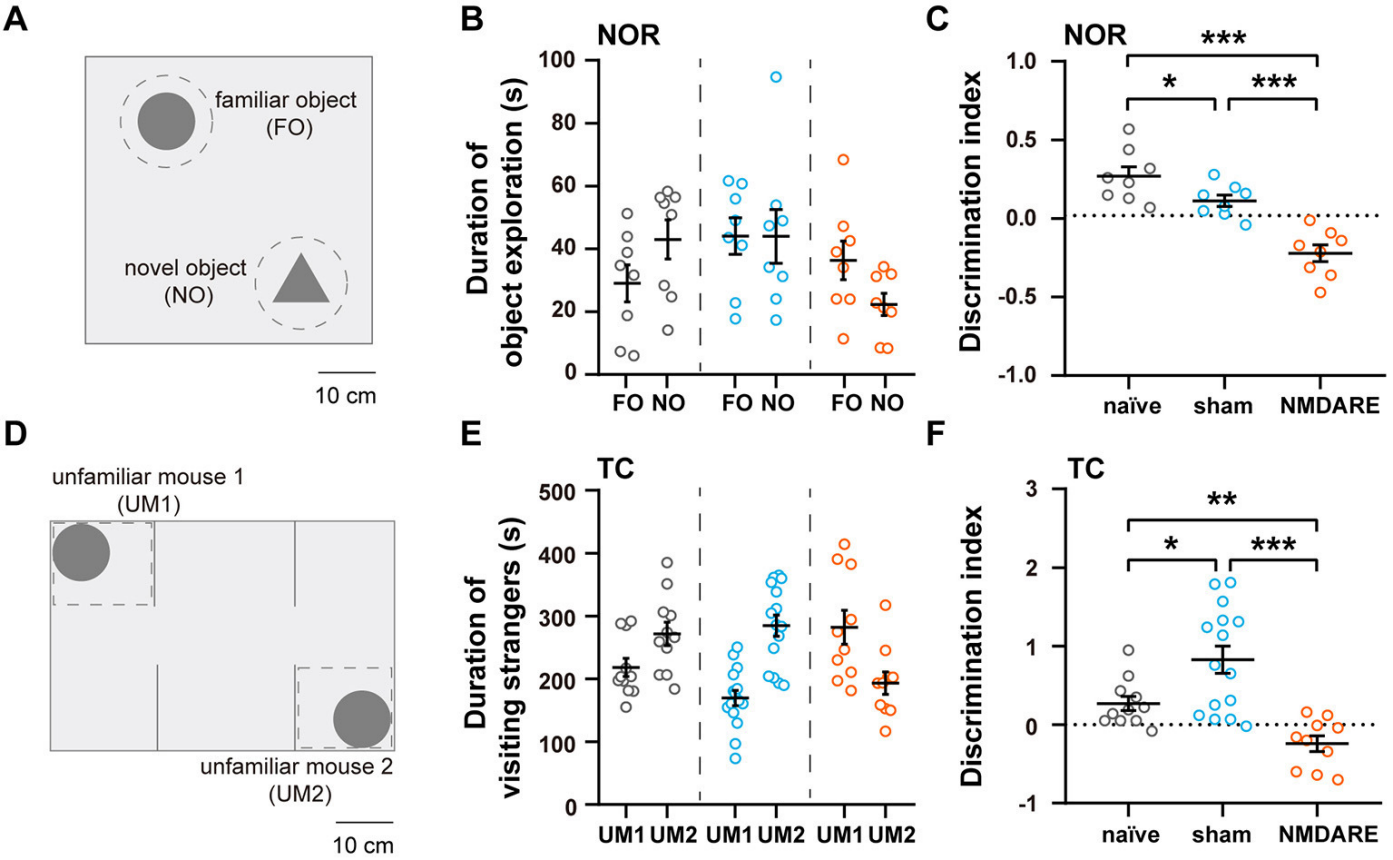
Cognition and sociability assessment via the novel object recognition and three chamber tests. **(A)** Diagram of experimental setup for the NOR test. Scale bar, 10 cm. **(B)** Total time spent exploring the familiar and novel object (naïve group, *n* = 8 mice; sham group, *n* = 8 mice; NMDARE group, *n* = 8 mice; gray circles, naïve group; blue circles, sham group; orange circles, NMDARE group). **(C)** DI-NOR values from the NOR test. **(D)** Diagram of experimental setup for the TC test. Scale bar, 10 cm. **(E)** Total time spent exploring each unfamiliar mouse during the TC test (naïve group, *n* = 11 mice; sham group, *n* = 15 mice; NMDARE group, *n* = 10 mice; gray circles, naïve group; blue circles, sham group; orange circles, NMDARE group). **(F)** DI-TC values from the TC test. *p < 0.05, **p < 0.01, ***p < 0.001, two-sample *t*-test.

The novel object recognition and the three chamber test were employed to assess the cognition and sociability of GluN1_356 − 385_-induced NMDRE mice, respectively (Figures 4A, D). Compared with the naïve group and the sham group, the NMDARE group spent more time exploring the familiar object instead of the novel object (Figure 4B). In the NOR test, the DI-NOR value corresponding to the NMDARE group decreased significantly and even reached negative values, suggesting that the NMDARE mice exhibited cognition impairment (Figure 4C). In the TC test, the NMDARE mice preferred to visit the first rather than the second unfamiliar mouse (Figure 4E). The DI-TC value of the NMDARE group became negative and was significantly decreased compared to that of both the naïve and the sham groups, demonstrating a reduction in sociability specific of the NMDARE mice (Figure 4F). Interestingly, the sham group showed a preference to interact with the second unfamiliar mouse, which resulted in a high DI-TC value (Figure 4F). These results confirm that the juvenile female NMDARE mice suffered a reduction in cognition and sociability.

### 3.4. Decreased LTP and fEPSP in the CA1 of juvenile female NMDARE mice

**Figure 5:**
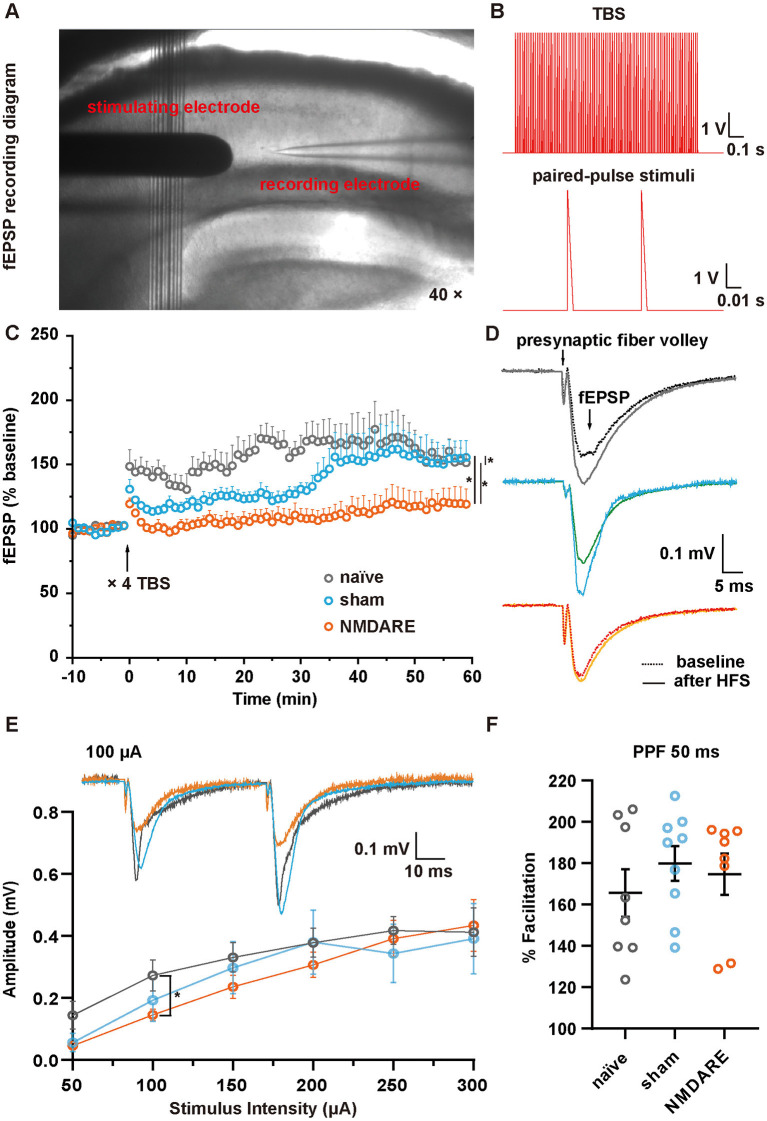
LTP and fEPSP in the hippocampal CA1 area of juvenile mice. **(A)** Image showing the location of the electrode during fEPSP recording in area CA1. **(B)** Theta-burst stimuli (TBS) and paired-pulse stimuli protocols. **(C)** Normalized amplitude of fEPSP. Following a 10-min baseline recording, four sequences of TBS were administered, followed by recording of fEPSPs for 1 h. Normalization was conducted according to the baseline value (naïve group, *n* = 6 slices from three mice; sham group, *n* = 9 slices from four mice; NMDARE group, *n* = 6 slices from three mice, *p < 0.05, two-way ANOVA. **(D)** Example traces of fEPSPs recorded before (dotted line) and after (full line) TBS. The stimuli artifacts underwent a smoothing process. **(E)** Paired-pulse stimuli at 50 ms intervals with a current range of 50–300 μA were recorded. The potential amplitude of the first pulse was measured to construct an I-O curve (naïve group, *n* = 8 slices from three mice; sham group, *n* = 9 slices from four mice; NMDARE group, *n* = 7 slices from four mice, *p < 0.05, two sample *t*-test, NMDARE vs. naive). Example traces of fEPSPs recorded in response to paired-pulse stimuli at a current intensity of 100 μA and an interval of 50 ms (top). **(F)** Statistical comparison of paired-pulse facilitation at a current intensity of 100 μA and an interval of 50 ms.

After all behavioral tests had been completed, a patch clamp experiment was performed in hippocampal slices obtained from naïve, sham and NMDARE mice to characterize cellular function in the CA1 area (Figure 5A). After receiving four sequences of TBS stimuli, the stimuli were recorded using paired-pulse recording protocol (Figure 5B). The results showed that the normalized fEPSP amplitude of the NMDARE group decreased significantly compared to that of the sham and naïve groups. There were also significant differences between the sham and naïve groups. Notably, NMDARE mice had more severe and complete LTP defects (Figures 5C, D). The I-O curve suggested that the absolute value of fEPSPs in the experimental group was significantly lower compared to that in the naïve group when stimulated by a 100 μA current, but there was no significant difference in absolute fEPSP amplitudes between the sham and NMDARE group (Figure 5E). No statistical difference among the three groups was evident for paired-pulse facilitation (PPF), measured as the amplitude of the last potential over the amplitude of the previous one, at a current intensity of 100 μA and an interval of 50 ms (Figure 5F). Considered together, the *in vitro* electrophysiological recording suggested that both synaptic transmission efficiency and synaptic plasticity of the hippocampal CA1 were impaired in NMDARE mice.

### 3.5. Epileptic susceptibility of juvenile female NMDARE mice

**Figure 6:**
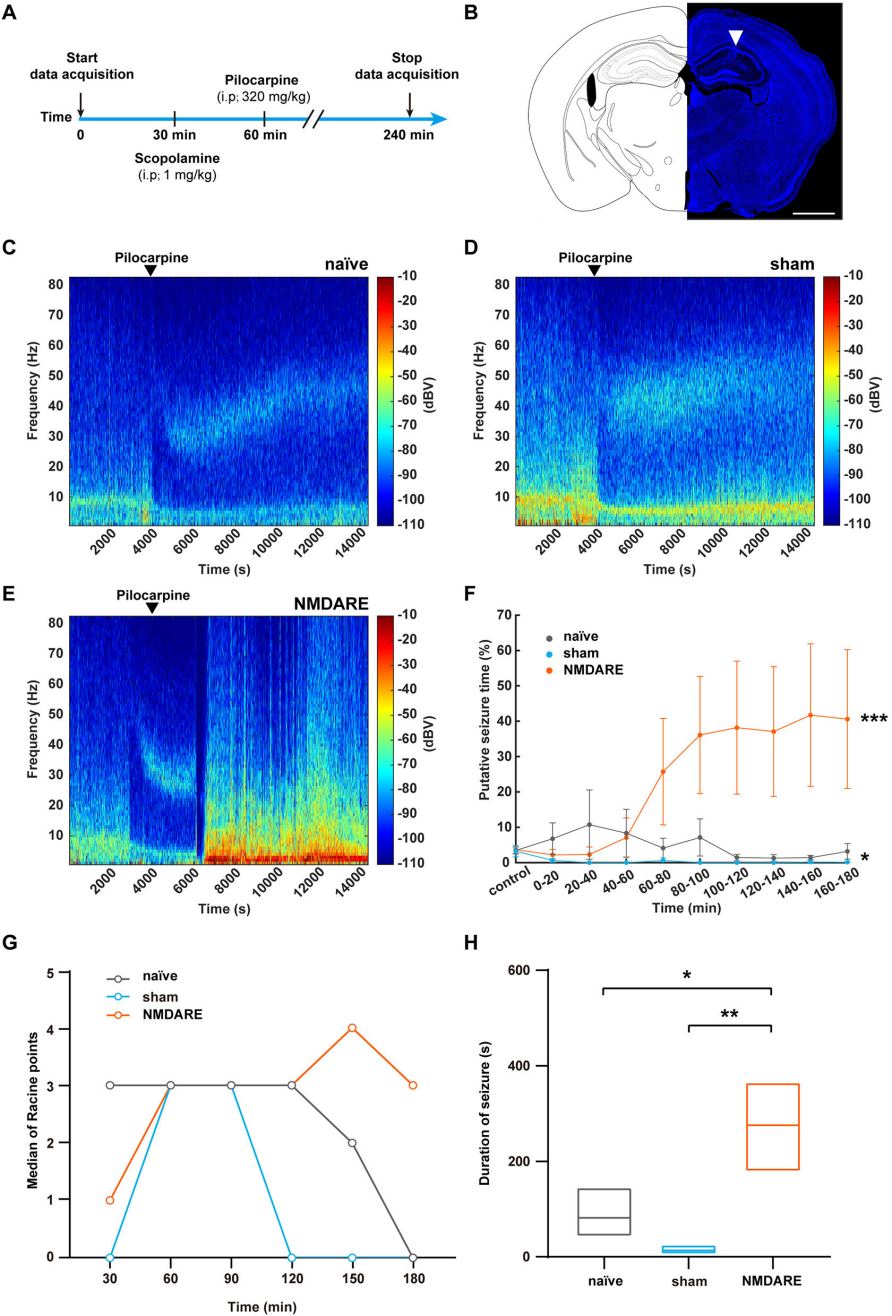
Evaluation of pilocarpine-induced seizures in NMDARE mice. **(A)** Diagram depicting the protocol used to elicit pilocarpine-induced seizures in naïve and NMDARE mice. **(B)** Representative trace of an electrode implanted in the dorsal hippocampus of NMDARE mice. Scale bar, 1 mm. **(C–E)** Representative spectrograms of LFPs recorded in response to pilocarpine administration in naïve **(C)**, sham **(D)**, and NMDARE mice **(E)**. **(F)** Putative seizure periods of naïve, sham, and NMDARE mice (naïve group, *n* = 6 mice; sham group, *n* = 4 mice, NMDARE group, *n* = 7 mice, two-way ANOVA, *p < 0.05, sham vs. naïve, ***p < 0.001, NMDARE vs. naïve). **(G)** Median Racine scale scores, scored in 30-min intervals. **(H)** Seizure duration (2–5 points) recorded for 3 h after pilocarpine injection (naïve group, *n* = 6 mice; sham group, *n* = 4 mice; NMDARE group, *n* = 7 mice). *p < 0.05, **p < 0.01, two-sample *t*-test.

No spontaneous seizure was observed in juvenile female NMDARE mice by monitoring of seizure behavior (*n* = 10) and recording the local field potential for 12 h (*n* = 2). Furthermore, to investigate whether the juvenile NMDARE mice are susceptible to epilepsy, we administered them pilocarpine to induce seizures, and both electrophysiological behavioral data were subsequently recorded (Figures 6A, B). We found that the LFP power spectrogram in naïve mice and sham mice showed decreased power (typical data shown in Figures 6C, D), whereas NMDARE mice showed increased LFP power, and four of the seven mice developed status epilepticus (typical data shown in Figure 6E). The statistical data also suggested that the NMDARE mice suffered sustained and more severe status epilepticus (Figure 6F). Thus, the results of local field potential showed that NMDARE mice are more likely to suffer higher intensity seizures compared with naïve and sham mice after being administered the same dose of pilocarpine. Similarly, the evaluation of seizure severity using the Racine scale showed that the duration of the seizures was longer, even though the naïve mice presented higher median Racine points in the first 30 min (Figures 6G, H). In summary, these results demonstrate that juvenile female NMDARE mice are susceptible to pilocarpine-induced seizures.

## 4. Discussion

Pediatric patients constitute up to ~40% of the total NMDAR encephalitis cases, which comprise ~80% of autoimmune encephalitis, and represent a neuropsychiatric syndrome that is different from adult NMDARE (Florance et al., 2009; Matricardi et al., 2016). Here, we generated a juvenile NMDARE mouse model, given that such a model has not been described in the literature. Undoubtedly, important progress has been made in the battle against human disease by using animal models (McGonigle and Ruggeri, 2014). In previous studies, adult NMDARE mice models were used to promote the understanding of the disease mechanism, the discovery of novel targets for treatment, and translational research (Wright et al., 2015; Rosch et al., 2018; Taraschenko et al., 2019; Wagnon et al., 2020; Ding et al., 2021; Steinke et al., 2023). Among them, active immunization was suggested to be preferable to passive immunization because the latter affected CSF and was therefore unable to reproduce the pathological process *in vivo*. Thus, we used the GluN1_356 − 385_ peptide targeting the amino-terminal domain of the GluN1 subunit, administering it via three separate injections within 9 days to develop a juvenile NMDARE mouse model based on active immunization.

First, we demonstrated that repeated GluN1_356 − 385_ peptide administration induced anti-NMDAR antibodies in juvenile female mice, which is consistent with previous studies in adult models (Wagnon et al., 2020; Ding et al., 2021). As PTX is known to damage the blood brain barrier, we used it to facilitate penetration of the peptide from the periphery, and this may have been the cause for the presence of IgG in the sham group (Munji et al., 2019).

NMDARE leads to a complex neuropsychiatric syndrome in patients, as well as in adult mouse models (Granerod et al., 2010; Xu et al., 2020; Zou et al., 2020). In our current study, we also evaluated behavioral traits, including locomotion, anxiety, cognition, and social behavior; we found that juvenile NMDARE mice exhibited significant cognitive and social impairment. Although the juvenile NMDARE mice exhibited reduced locomotion compared to naïve mice, the NMDARE group did not display significant differences in anxiety-like behavior or locomotion deficiency when compared to the sham group. Since the CFA is applied to promote inflammatory response, we used it to enhance the immune response to the GluN1 peptide (Fontes et al., 2017). In other studies, CFA was used to induce chronic pain in rodents, causing movement deficits and mechanical hypersensitivity (Liu et al., 2015; Sheahan et al., 2017; Ferdousi et al., 2019; Pitzer et al., 2019). Thus, we could not determine whether the locomotion and anxiety levels in sham and NMDARE group were caused by chronic pain.

To confirm that the juvenile NMDARE mice suffer from neuronal dysfunction in typical brain regions, we investigated their electrophysiological characteristics in the hippocampus *in vitro*. We found that LTP in the hippocampal CA1 area of NMDARE mice was significantly impaired compared to that in both naïve and sham mice. According to the literature, subcutaneous injection of CFA can lead to chronic pain accompanied with memory deficits in which the hippocampus plays an important regulatory role (Liu et al., 2015; Ferdousi et al., 2019; Li et al., 2019). Since the sham group was injected with the control drug containing CFA, the degree of LTP in sham mice was less than that of naïve mice, but unsurprisingly the LTP defect of the NMDARE mice was more serious and extensive. LTP in the hippocampus is known to play an important role in learning and memory, and therefore this result is consistent with the cognition deficiency displayed by NMDARE mice. Since the pathogenesis of NMDARE involves the destruction of NMDARs, we hypothesized that the decrease of LTP in NMDARE mice might have been caused by the binding of specific antibodies to NMDARs in the postsynaptic membrane, leading to a decrease in the total number and in the function of the receptor. It has previously been reported that surface trafficking of NMDARs contributes to the modulation of synaptic function and information processing. The I-O curve suggests that synaptic function is impaired in NMDARE mice compared to naïve mice. The data from the sham group implied that the operation procedure may also partly contribute to the results obtained. The PPF results showed no significant differences among the three groups, suggesting that there are no differences at the presynaptic level and that the functional impairment is therefore more likely to be a postsynaptic effect. Furthermore, regulation of functional α-amino-3-hydroxy-5-methyl-4-isoxazole-propionic acid receptors (AMPARs) also plays a key role in synaptic plasticity (Palmer et al., 2005). NMDAR trafficking can regulate AMPAR trafficking and AMPAR-mediated LTP (Yang et al., 2022). Therefore, after NMDARs are neutralized by specific antibodies in our model animals, the decreased NMDAR function may also significantly reduce LTP and affect learning and memory by disrupting AMPAR trafficking in turn.

Given that we did not observe spontaneous seizures in our juvenile NMDARE mice, their sensitivity to pharmacology-induced epilepsy was tested. We demonstrated that the juvenile NMDARE mice were susceptible to epilepsy, which is consistent with the clinical observation that seizures are more frequent in pediatric patients (Titulaer et al., 2013; Zhang et al., 2022). Taken together, our results confirm that the GluN1_356 − 385_ peptide specifically induced anti-NMDAR antibodies, resulting in impaired cognition and sociability; decreased synaptic transmission efficiency and synaptic plasticity in the hippocampus; and promoted susceptibility to epilepsy in our juvenile NMDARE mice. Despite the use of PTX and CFA, our results are in agreement with the clinical manifestations previously described in pediatric patients. The absence of timely diagnosis and treatment in these patients may affect the development of the nervous system and result in lifelong effects. Therefore, the proposed model could help accelerate research on juvenile NMDARE and derive in benefits for the patients.

## 5. Conclusions

In view of the clinical differences between juvenile and adult anti-NMDAR encephalitis patients, and the lack of appropriate animal models for the former, we developed a mouse model for juvenile NMDARE by active immunization with the GluN1_356 − 385_ peptide. The juvenile female NMDARE mouse model generated anti-NMDAR autoantibodies *in vivo* and displayed cognitive impairment and reduced sociability, which are similar to the symptoms described in pediatric patients. Regarding cellular function, both synaptic transmission efficiency and synaptic plasticity were impaired. Moreover, the model showed enhanced susceptibility to epilepsy. The juvenile female NMDARE mouse model may accelerate research on the pathological mechanisms and treatments for anti-NMDAR encephalitis, thus benefiting both adult and pediatric patients.

## Data availability statement

The original contributions presented in the study are included in the article/supplementary material, further inquiries can be directed to the corresponding authors.

## Ethics statement

The animal study was approved by the Ethics Committee for Animal Research from the Shenzhen Institute of Advanced Technology, Chinese Academy of Sciences (SIAT-IACUC-220217-NS-ZC-A2121). The study was conducted in accordance with the local legislation and institutional requirements.

## Author contributions

CZ, SH, CS, and SL were responsible for the conception of the study, the design of the experiments, and the interpretation of the data. SH, QZ, CS, YC, JH, and LL acquired the data. SH, CS, GW, QZ, and JH analyzed the data. CZ, CS, SH, and QZ wrote the manuscript. QS, JL, XM, and YL commented on the manuscript. XM made great contribution to revision. All authors contributed to the manuscript and approved the submitted version.
